# Palmitoyltransferase DHHC13 controls lysosomal cholesterol egress through palmitoylating Niemann-Pick C1

**DOI:** 10.1093/lifemeta/loag028

**Published:** 2026-09-10

**Authors:** Xiao-Ting Men, Xiao-Yu Bai, Yin-Yue Zhao, Yu-Qing Huang, Ding-Yuan Huo, Yu-Xiao Jiang, Ming-Feng Xia, Haobin Ye, Linzhang Huang, Xingrong Du, Hong-Jie Qian, Shuai Chen, Qing Shi, Tong-Jin Zhao

**Affiliations:** State Key Laboratory of Genetics and Development of Complex Phenotypes, Shanghai Key Laboratory of Metabolic Remodeling and Health, Institute of Metabolism and Integrative Biology, Zhongshan Hospital, Fudan University, 2005 Songhu Road, Shanghai 200438, China; State Key Laboratory of Genetics and Development of Complex Phenotypes, Shanghai Key Laboratory of Metabolic Remodeling and Health, Institute of Metabolism and Integrative Biology, Zhongshan Hospital, Fudan University, 2005 Songhu Road, Shanghai 200438, China; State Key Laboratory of Genetics and Development of Complex Phenotypes, Shanghai Key Laboratory of Metabolic Remodeling and Health, Institute of Metabolism and Integrative Biology, Zhongshan Hospital, Fudan University, 2005 Songhu Road, Shanghai 200438, China; State Key Laboratory of Genetics and Development of Complex Phenotypes, Shanghai Key Laboratory of Metabolic Remodeling and Health, Institute of Metabolism and Integrative Biology, Zhongshan Hospital, Fudan University, 2005 Songhu Road, Shanghai 200438, China; State Key Laboratory of Genetics and Development of Complex Phenotypes, Shanghai Key Laboratory of Metabolic Remodeling and Health, Institute of Metabolism and Integrative Biology, Zhongshan Hospital, Fudan University, 2005 Songhu Road, Shanghai 200438, China; Department of Endocrinology, Zhongshan Hospital, Fudan University, 180 Fenglin Road, Shanghai 200032, China; Department of Endocrinology, Zhongshan Hospital, Fudan University, 180 Fenglin Road, Shanghai 200032, China; State Key Laboratory of Genetics and Development of Complex Phenotypes, Shanghai Key Laboratory of Metabolic Remodeling and Health, Institute of Metabolism and Integrative Biology, Zhongshan Hospital, Fudan University, 2005 Songhu Road, Shanghai 200438, China; State Key Laboratory of Genetics and Development of Complex Phenotypes, Shanghai Key Laboratory of Metabolic Remodeling and Health, Institute of Metabolism and Integrative Biology, Zhongshan Hospital, Fudan University, 2005 Songhu Road, Shanghai 200438, China; State Key Laboratory of Genetics and Development of Complex Phenotypes, Shanghai Key Laboratory of Metabolic Remodeling and Health, Institute of Metabolism and Integrative Biology, Zhongshan Hospital, Fudan University, 2005 Songhu Road, Shanghai 200438, China; Drug Clinical Trial Center, Shanghai Xuhui Central Hospital/Xuhui Hospital, Fudan University, Shanghai Engineering Research Center of Phase I Clinical Research and Quality Consistency Evaluation for Drugs, 366 Longchuan North Road, Shanghai 200237, China; State Key Laboratory of Pharmaceutical Biotechnology and MOE Key Laboratory of Model Animal for Disease Study, Model Animal Research Center, School of Medicine, Nanjing University, 12 Xuefu Road, Pukou District, Nanjing, Jiangsu 210008, China; State Key Laboratory of Genetics and Development of Complex Phenotypes, Shanghai Key Laboratory of Metabolic Remodeling and Health, Institute of Metabolism and Integrative Biology, Zhongshan Hospital, Fudan University, 2005 Songhu Road, Shanghai 200438, China; State Key Laboratory of Genetics and Development of Complex Phenotypes, Shanghai Key Laboratory of Metabolic Remodeling and Health, Institute of Metabolism and Integrative Biology, Zhongshan Hospital, Fudan University, 2005 Songhu Road, Shanghai 200438, China; State Key Laboratory of Metabolic Dysregulation & Prevention and Treatment of Esophageal Cancer, Innovation Center of Basic Research for Metabolic-Associated Fatty Liver Disease, Ministry of Education of China, Tianjian Laboratory of Advanced Biomedical Sciences, Academy of Medical Sciences, Zhengzhou University, 100 Kexue Avenue, Zhengzhou, Henan 450001, China

**Keywords:** DHHC13, palmitoylation, NPC1, lysosomal cholesterol egress, Niemann-Pick disease type C

## Abstract

Protein S-palmitoylation provides a key regulatory mechanism for diverse cellular activities. Although palmitoylated proteins are frequently observed in cholesterol-enriched membrane microdomains, how protein palmitoylation reciprocally regulates cholesterol metabolism remains elusive. Here, we identify DHHC13 as a critical regulator linking protein palmitoylation to lysosomal cholesterol egress. Through an shRNA screen, we found that lack of *DHHC13* led to pronounced lysosomal cholesterol accumulation. Mechanistically, DHHC13 directly palmitoylated Niemann-Pick C1 (NPC1). Loss of palmitoylation disrupted NPC1 association with lysosomes and caused its degradation, thereby impairing lysosomal cholesterol egress. *Dhhc13*^−/−^ mice phenocopied *Npc1*^−/−^ mice, displaying cholesterol deposition in the brain, liver, spleen, and kidney, along with significant Purkinje cell loss. Furthermore, two clinically relevant DHHC13 mutants (Q173H and R451Q) associated with neurodegenerative disorders showed diminished catalytic activity and failed to restore NPC1 palmitoylation or lysosomal cholesterol egress. Collectively, our findings uncover DHHC13 as a critical regulator of lysosomal cholesterol metabolism via NPC1 palmitoylation, providing mechanistic insights into cholesterol-related neurological disorders.

## Introduction

Protein S-palmitoylation is a reversible post-translational lipid modification with the attachment of palmitoyl groups to thiol groups of cysteine residues [1]. The reaction is catalyzed by a family of Asp-His-His-Cys (DHHC) motif containing palmitoyltransferases and removed by a group of depalmitoylases [2–7]. Dynamic protein palmitoylation plays a pivotal role in cellular activities by regulating protein trafficking, subcellular localization, protein stability, and protein−protein interaction [8–12]. Although palmitoylated proteins are frequently enriched in cholesterol-rich membrane microdomains, and cholesterol is required for their residency within these domains [13–16], whether and how protein palmitoylation reciprocally regulates cholesterol metabolism remains elusive.

Cholesterol is an essential nutrient for cellular homeostasis. Low-density lipoprotein (LDL) is a major source of cholesterol via the LDL receptor (LDLR)-mediated endocytosis [17]. Internalized LDL particles are delivered to lysosomes, where cholesteryl esters are hydrolyzed into free cholesterol and fatty acids [18, 19]. The lysosomal egress of free cholesterol is cooperatively mediated by Niemann‑Pick C1 (NPC1) and Niemann‑Pick C2 (NPC2): NPC2 captures and transfers the sterol to NPC1, which then transports it across the lysosomal membrane [20–23]. Loss-of-function mutations in either NPC1 or NPC2 cause Niemann-Pick disease type C (NPC), a severe lysosomal storage disorder characterized by massive cholesterol and lipid accumulation in lysosomes, ultimately leading to progressive neurodegeneration [24, 25]. Notably, NPC1 has been reported to be a palmitoylated protein, and this modification is required for its cholesterol transporting activity [25–27]. However, previous findings remain inconsistent regarding the exact palmitoylation sites on NPC1 and the specific impact of palmitoylation on its lysosomal localization, leaving the identity of the responsible palmitoyltransferase and its regulatory mechanism unclear.

Zinc finger DHHC‑type palmitoyltransferase 13 (DHHC13), also known as huntingtin‑interacting protein 14 (HIP14)-related protein, is a member of the DHHC family enzymes [28]. DHHC13 is widely expressed across various tissues and primarily localized to the Golgi apparatus and cytosolic vesicles [29]. DHHC13 palmitoylates a diverse range of substrates and is implicated in skin barrier integrity [30], hair growth [31], skeletal development [32], mitochondrial function [33, 34], autophagy [35, 36], and cancer progression [37]. Mice bearing a loss-of-function mutation in *Dhhc13* (R425X) exhibit small size and shortened life-span, along with alopecia, severe osteoporosis, systemic amyloidosis, muscle wasting, and neutrophilia [38]. The role of DHHC13 in cholesterol metabolism has not been reported.

Here, to address the role of protein palmitoylation in cholesterol metabolism, we identified DHHC13 as a critical regulator of lysosomal cholesterol egress. We demonstrated that DHHC13 palmitoylated NPC1, which was required for the lysosomal association of NPC1 and its stability. Importantly, the mouse NPC1-cysteine‑1167‑to‑tyrosine (C1167Y) mutant, corresponding to the disease-associated human NPC1-C1168Y variant, exhibited markedly reduced palmitoylation and protein stability and failed to rescue lysosomal cholesterol egress in NPC1-deficient cells. Furthermore, *Dhhc13*^−/−^ mice phenocopied *Npc1*^−/−^ mice, displaying pronounced cholesterol deposition in the brain, liver, spleen, and kidney, accompanied by a significant loss of Purkinje cells. We further identified two clinically relevant DHHC13 mutants (Q173H and R451Q) associated with neurological disorders; both mutants were unable to restore NPC1 palmitoylation or alleviate lysosomal cholesterol accumulation in *DHHC13*^−/−^ cells. Collectively, our findings uncover DHHC13 as a key regulator in lysosomal cholesterol egress and provide novel insights into the regulation of cholesterol metabolism through protein palmitoylation.

## Results

### DHHC13 is required for cholesterol egress from lysosomes

To investigate the potential role of protein palmitoylation in intracellular cholesterol trafficking, we examined the effect of 2-bromopalmitate (2-BP), a pan-DHHC inhibitor, on lysosomal cholesterol egress in LDL-treated HeLa cells. Filipin staining revealed that 2-BP treatment increased intracellular cholesterol levels by approximately 40% (Fig. 1a and b), suggesting a potential involvement of protein palmitoylation in lysosomal cholesterol egress. To further validate this observation, we employed amphotericin B (AmB), which kills cells by irreversibly binding to plasma membrane cholesterol, to assess lysosomal cholesterol egress [39]. While AmB induced time-dependent cell death under normal conditions with a half-life of about 2 h, this effect was largely attenuated in cells pretreated with 2-BP (Fig. 1c and d), further supporting a role for protein palmitoylation in lysosomal cholesterol transport.

**Figure 1:**
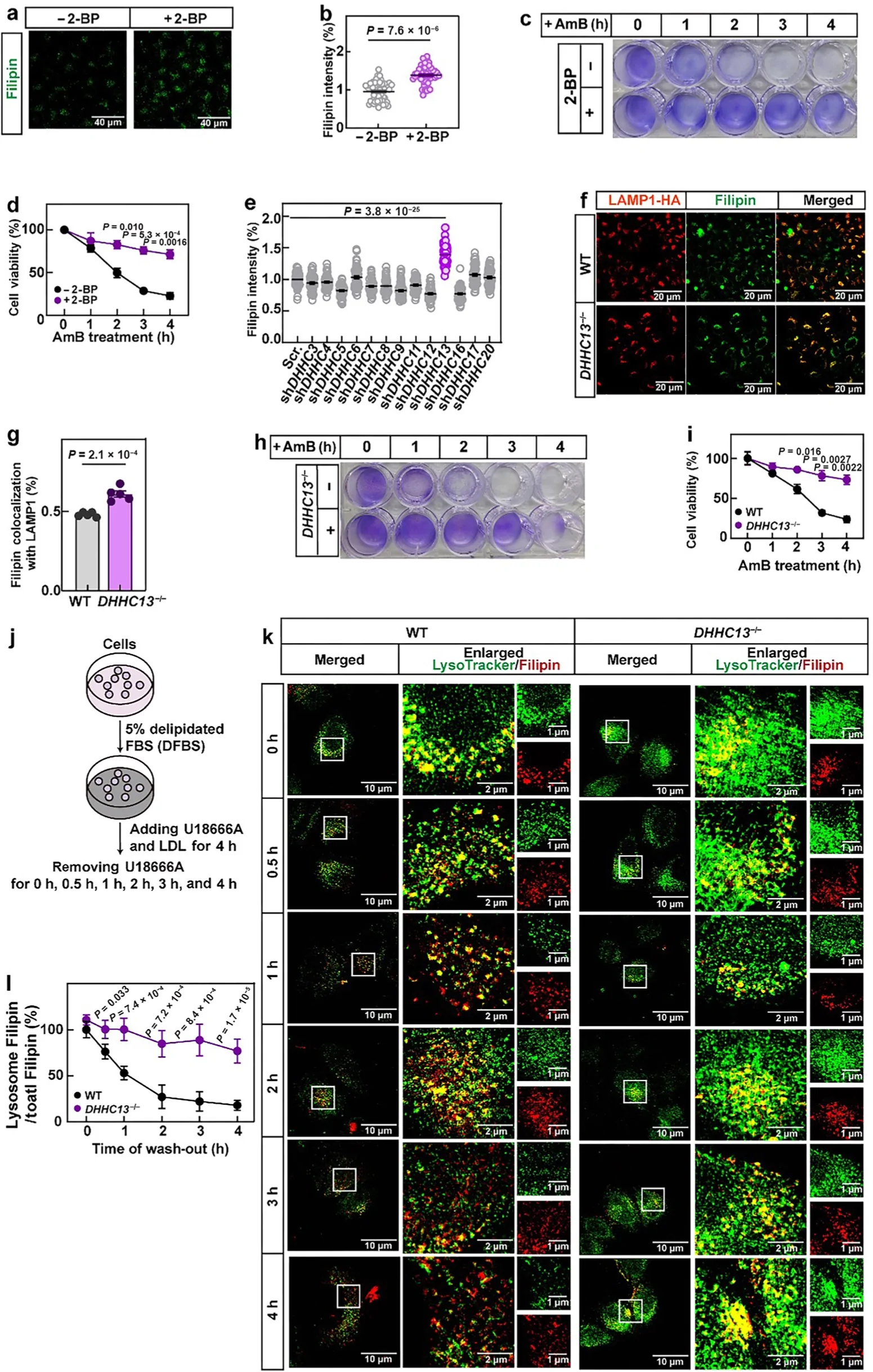
DHHC13 is required for cholesterol egress from lysosomes. (a and b) Filipin staining and quantification of mean Filipin fluorescence intensity in HeLa cells treated with 2-BP. On day 0, HeLa cells were set up at 1 × 10^5^ cells per 3.5-cm dish. On day 1, cells were treated with 10% BSA or 40 μmol/L 2-BP for 12 h. On day 2, cells were cultured in DMEM supplemented with 5% DFBS for 12 h, followed by the addition of 20 μg/mL LDL for an additional 12 h. On day 3, cells were harvested for Filipin staining analysis. Quantification of the mean Filipin fluorescence intensity was performed using Image J software, with 50 cells randomly selected across five independent fields of view. (c and d) Cell viability assay with AmB selection. On day 0, HeLa cells were set up as in (a), and subjected to the AmB-selection protocol as described in the Methods. Cells were treated with AmB in the presence or absence of 2-BP for the indicated durations. Representative images of the cell viability assay are shown in (c). Quantification of cell viability is shown in (d; *n *= 3). (e) Filipin staining in HeLa cells with indicated shRNA knockdown. On day 0, HeLa cells were set up and treated as in (a). On day 1, HeLa cells were transduced with indicated shRNA, and selected with puromycin. On day 5, cells were cultured in DMEM supplemented with 5% DFBS for 12 h and 20 μg/mL LDL was added for 12 h before being harvested for immunofluorescence. Quantification was performed as in (b). (f and g) Co-immunostaining of Filipin-positive cholesterol with LAMP1-labeled lysosomes. On day 0, WT and *DHHC13*^−/−^ HeLa cells were set up. On day 2, cells were transfected with 2 μg of LAMP1-HA/pCDH-puro. On day 3, cells were cultured in DMEM supplemented with 5% DFBS for 12 h, followed by the addition of 20 μg/mL LDL for an additional 12 h. Cells were harvested for immunostaining analysis with Filipin. The proportion of Filipin-positive cholesterol signals overlapping with LAMP1-positive lysosomal structures was quantified using Image J software (*n *= 5). (h and i) Crystal violet staining of WT and *DHHC13*^−/−^ HeLa cells. On day 0, HeLa cells were set up and treated as in (f). On day 1, cells were treat as in (c). On day 3, cells were stained with crystal violet, and quantification was performed as in (d; *n *= 3). (j–l) Filipin and LysoTracker staining in U18666A-treated HeLa cells. On day 0, HeLa cells were set up as in (a), and subjected to the AmB-selection protocol as described in the Methods. Cells were washed to remove U18666A (j) and then harvested for fluorescent staining with Filipin and LysoTraker (l). Quantification of lysosome Filipin/total Filipin is shown in (l; *n *= 5). Each value represents mean ± SEM (b, d, e, g, i, and l). *P*-values were calculated from two-tailed unpaired *t*-tests.

To identify the specific DHHC enzyme involved, we performed an shRNA-based screen by individually knocking down 13 relatively abundant DHHC family members in HeLa cells and assessing intracellular cholesterol content. Knockdown of *DHHC13* increased Filipin staining intensity by approximately 50% (Fig. 1e; Supplementary Fig. S1a and b). Co-immunostaining with lysosome‑associated membrane protein 1 (LAMP1) confirmed that the accumulated cholesterol was localized to lysosomes (Fig. 1f and g). Furthermore, knockout of *DHHC13* significantly delayed AmB-induced cell death (Fig. 1h and i).

To further consolidate the results, we treated LDL-loaded wild-type (WT) and *DHHC13*^−/−^ cells with the U18666A compound to block lysosomal cholesterol efflux, and monitored the process after removal of U18666A. In WT cells, the Filipin signal (indicative of cholesterol) dissociated from LysoTracker in a time-dependent manner. By 2 h, about 75% of the Filipin signals were dissociated from lysosomes and slowly decreased afterwards (Fig. 1j–l). However, in *DHHC13*^−/−^ cells, over 80% of the cholesterol signals remained in the lysosomes in the 4-h period after removal of U18666A (Fig. 1j–l). These results indicate that DHHC13 plays an essential role in lysosomal cholesterol efflux.

### DHHC13 palmitoylates NPC1 and controls its lysosomal localization and stability

We next sought to identify the substrate of DHHC13 in mediating cholesterol transport. Palmitoylated proteins were isolated from WT and *DHHC13*^−/−^ HEK-293T cells using Acyl-Resin Assisted Capture (Acyl-RAC) and subjected to mass spectrometry analysis (Fig. 2a). Among the potential substrates identified, NPC1 caught our attention due to its well-established role in lysosomal cholesterol egress [20, 40, 41]. Six peptides corresponding to NPC1 were detected in WT cells, whereas none was identified in *DHHC13*^−/−^ cells (Fig. 2b). We confirmed these findings by Acyl-RAC followed by immunoblotting, which showed that knockout of *Dhhc13* dramatically decreased both total (approximately 45%) and palmitoylated (approximately 90%) NPC1 levels (Fig. 2c–e). Even after normalization of total NPC1, palmitoylated NPC1 remained significantly reduced (approximately 75%) in *DHHC13*^−/−^ cells (Fig. 2f and g). Cycloheximide (CHX) chase assays revealed that loss of DHHC13 markedly shortened the half-life of NPC1 (approximately 12 h vs. approximately 24 h in control cells; Fig. 2h and i). Moreover, NPC1 degradation in *Dhhc13* knockout cells was blocked by bafilomycin A1 (Fig. 2j), indicating a lysosomal degradation pathway.

**Figure 2:**
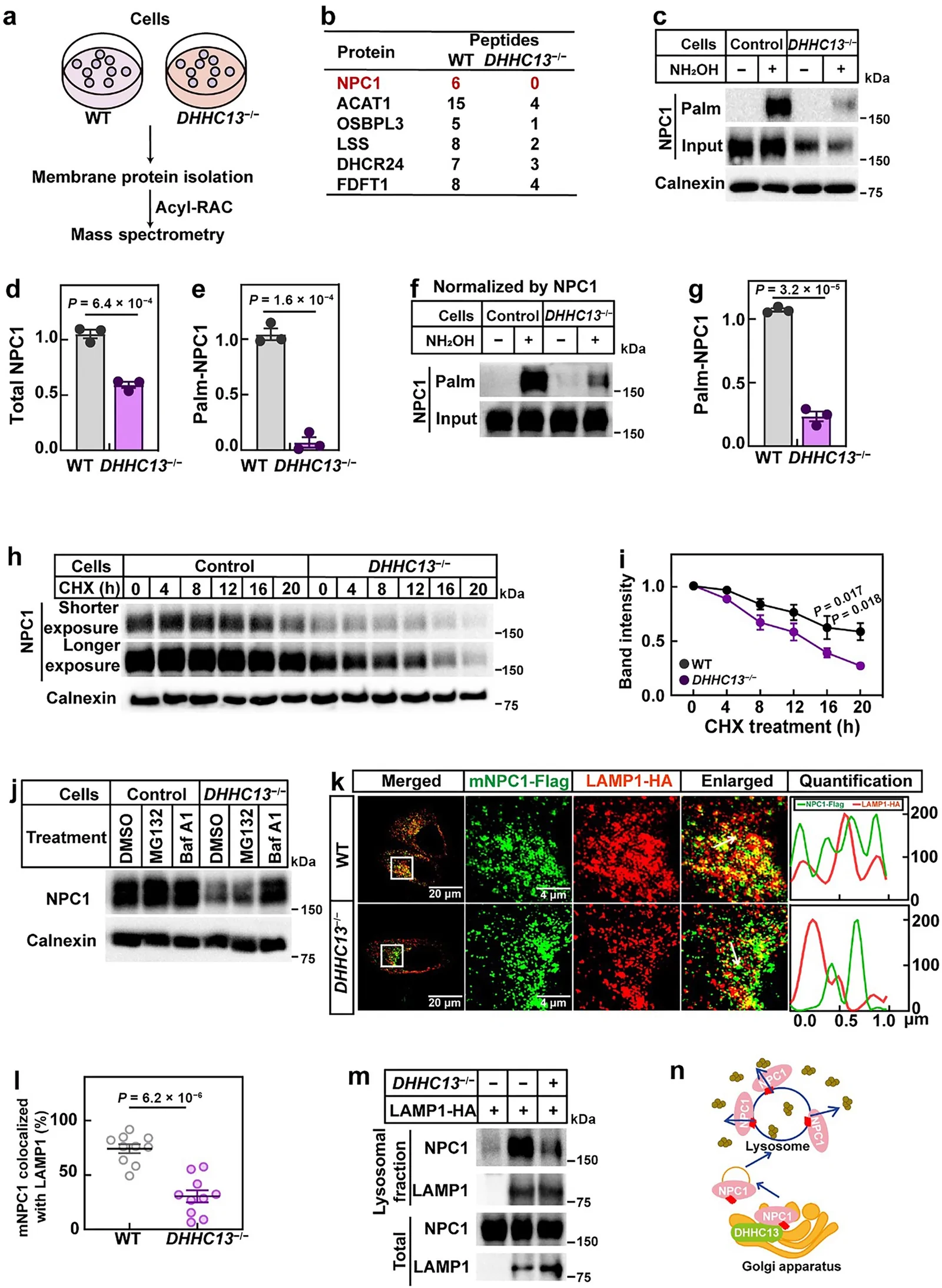
DHHC13 palmitoylates NPC1 and controls its lysosomal localization and stability. (a and b) Acyl-RAC assay coupled with mass spectrometry for identification of palmitoylated proteins. On day 0, membrane fractions were prepared from WT and *DHHC13*^−/−^ cell samples. Palmitoylated proteins were isolated by Acyl-RAC assay, and subjected to mass spectrometry analysis (a). The number of detected peptides for the identified proteins is listed in (b). (c–g) Western blot analysis of palmitoylated NPC1 in WT and *DHHC13*^−/−^ cells by Acyl-RAC assay. Palmitoylated proteins were isolated from WT and *DHHC13*^−/−^ HEK-293T cells by Acyl-RAC analysis and analyzed by Western blot using an antibody against endogenous NPC1. Samples were normalized to NPC1 levels (f). Band intensities of NPC1 (d and e) and normalized palmitoylated NPC1 (g) were quantified using ImageJ (*n *= 3), with Calnexin serving as an internal control. (h and i) CHX chase assay for NPC1 protein stability. WT and *DHHC13*^−/−^ HEK-293T cells were set up. On day 1, cells were treated with CHX (50 μg/mL) for the indicated time before harvesting for Western blot analysis (h). Band intensities were quantified using ImageJ (I; *n *= 3). (j) Effects of MG132 and bafilomycin A1 on NPC1 protein levels. WT and *DHHC13*^−/−^ HEK-293T cells were set up. On day 1, cells were treated with MG132 (20 μg/mL) or bafilomycin A1 (100 nmol/L) for 12 h. Proteins were extracted and subjected to Western blot analysis. (k and l) Fluorescence intensity profiles of NPC1-Flag and LAMP1-HA colocalization. WT and *DHHC13*^−/−^ HeLa cells were set up. On day 1, cells were transfected with 2 μg of LAMP1-HA/pCDH-puro and mNPC1-FLAG/pCDH-puro. On day 3, cells were harvested for immunostaining. (k) Fluorescence intensity profiles of NPC1-Flag and LAMP1-HA signals along the indicated lines generated using ZEN software. (l) The percentage of NPC1-Flag-positive area overlapping with LAMP1-HA-positive lysosomal structures from 10 cells quantified using ImageJ software. (m) Lysosomal fraction analysis of NPC1 and LAMP1-HA in WT and *DHHC13*^−/−^ cells. On day 0, WT and *DHHC13*^−/−^ HEK-293T cells were set up. On day 1, cells were transfected with 2 μg of LAMP1-HA/pCDH-puro. On day 3, cells were harvested for lysosomal fraction isolation and Western blot analysis. (n) Working model of DHHC13-mediated NPC1 palmitoylation in lysosomal trafficking. DHHC13 palmitoylates NPC1, which facilitates the trafficking of NPC1 from the Golgi to the lysosome. Lysosomal localization of NPC1 is required for its function in lysosomal cholesterol transport. Each value represents mean ± SEM of three samples (d, e, g, i, and l). *P*-values were calculated from two-tailed unpaired *t*-tests.

We next examined the effect of DHHC13 on the subcellular localization of NPC1. Immunofluorescence analysis revealed that the colocalization of NPC1 with the lysosomal marker LAMP1 decreased from 75% to 35% in *DHHC13*^−/−^ cells (Fig. 2k and l). Furthermore, biochemical isolation of lysosomes revealed that lysosomal content of NPC1 was dramatically decreased in *DHHC13*-knockout cells (Fig. 2m).

Together, these results support a model in which DHHC13-mediated palmitoylation promotes the trafficking and stable lysosomal localization of NPC1, thereby facilitating cholesterol egress from lysosomes (Fig. 2n).

### NPC1 palmitoylation is required for its stability and activity in lysosomal cholesterol egress

NPC1 has previously been reported to be palmitoylated, but its modification sites remain controversial. One study identified cysteine‑97 (Cys97) as a palmitoylation site of NPC1 [26], while the other identified cysteine‑799 (Cys799) and cysteine‑800 (Cys800) [27]. To resolve this discrepancy, we generated cysteine‑97‑to‑­serine (C97S) and cysteine‑799‑to‑serine/cysteine‑800‑to‑serine (C799S/C800S) mutants. The C97S mutant did not exhibit reduced NPC1 palmitoylation (Supplementary Fig. S2a), whereas the C799S/C800S mutation caused a marked but incomplete reduction (Supplementary Fig. S2b), supporting Cys799 and Cys800 as major palmitoylation sites while suggesting that additional residues may influence this modification.

To look for other potential palmitoylation sites, we noticed that the disease-associated C1168Y [in mouse cysteine‑1167 (Cys1167)] variant reduces NPC1 abundance [42, 43]. As cysteine‑1160 (Cys1160) is the vicinity, we generated the C1160S and C1167S mutants and found that these two mutants both exhibited markedly reduced palmitoylation (Fig. 3a). When Cys799, Cys800, Cys1160, and Cys1167 were mutated to serine (the 4CS mutant), the palmitoylation of NPC1 was largely abolished (Fig. 3a). Overexpression of DHHC13 increased the palmitoylation of WT, but not the 4CS mutant of NPC1, in HEK-293T cells (Supplementary Fig. S2c). The 4CS mutant also displayed a shortened half-life, and its degradation was largely blocked by bafilomycin A1 (Fig. 3b and c; Supplementary Fig. S2e).

**Figure 3:**
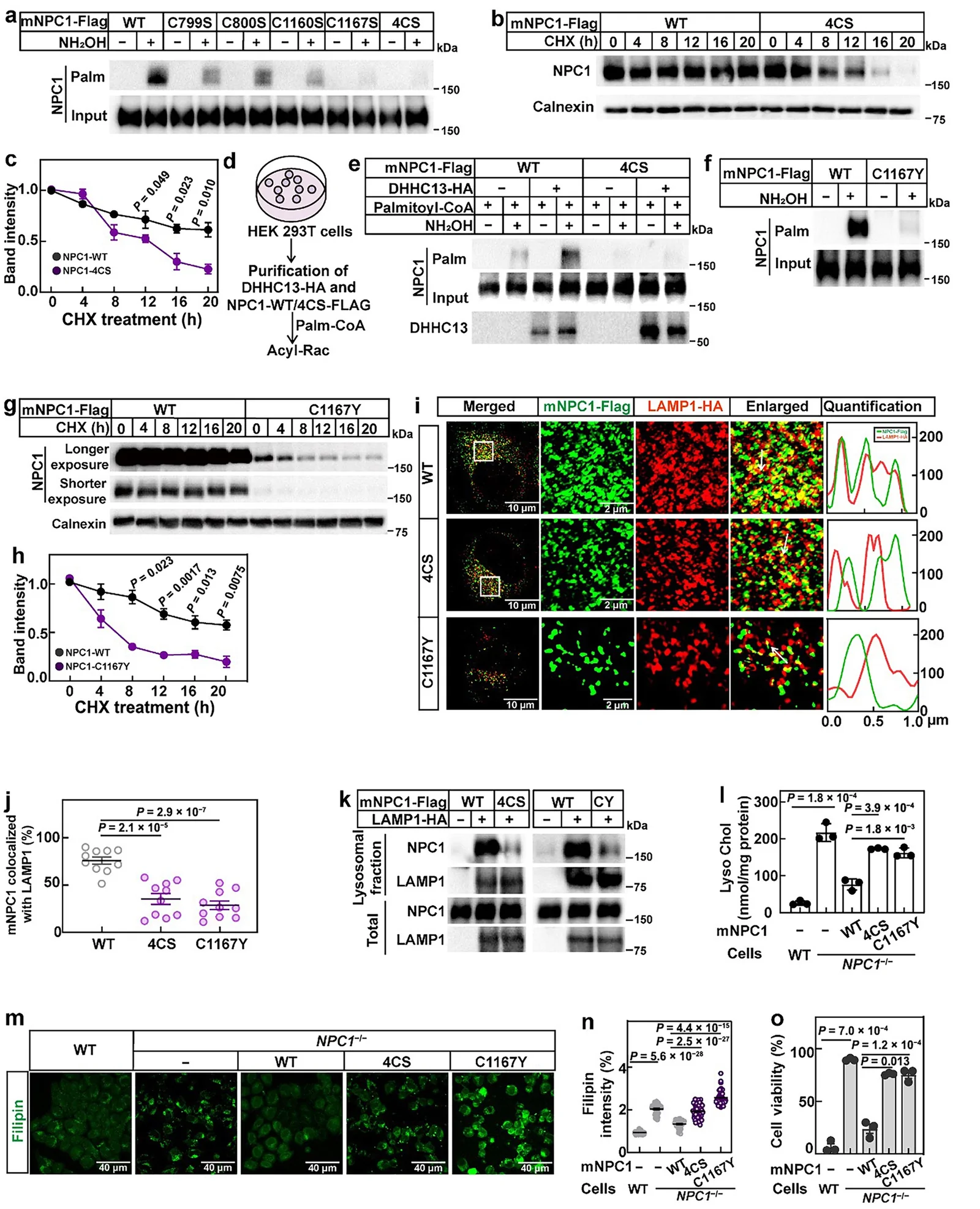
Palmitoylation of NPC1 is required for its lysosomal localization and cholesterol egress. (a) Acyl-RAC assay of WT and palmitoylation-deficient NPC1 mutants. On day 0, HEK-293T cells were set up at 2 × 10^5^ cells per 6-cm dish. On day 2, cells were transfected with 2 μg of either WT, C799S, C800S, C1160S, C1167S, or the palmitoylation-deficient C799S/C800S/C1160S/C1167S mutant (NPC1-4CS)-FLAG/pCDH-puro. On day 3, total protein was extracted for the Acyl-RAC assay and subjected to Western blot analysis. (b and c) CHX chase assay of WT and NPC1-4CS mutant. HEK-293T cells were set up and transfected with the indicated plasmids as in (a). On day 3, cells were cultured in complete medium, and treated with CHX (50 μg/mL) for the indicated time before harvesting for Western blot analysis (b). Band intensities were quantified with ImageJ using Calnexin as an internal control (c; *n *= 3). (d and e) *In vitro* palmitoylation assay of NPC1-WT and NPC1-4CS by DHHC13-HA. HEK-293T cells were set up and transfected with the indicated plasmids. On day 3, cells were harvested and DHHC13-HA and NPC1-WT/4CS-FLAG proteins were purified for the *in vitro* palmitoylation protocol. (f) Acyl-RAC assay of WT and C1167Y mutant NPC1. On day 0, HEK-293T cells were set up and transfected with 2 μg of either WT mNPC1-FLAG/pCDH-puro or the mouse C1167Y mutant (corresponding to human C1168Y) mNPC1-FLAG/pCDH-puro. On day 3, cells were harvested and palmitoylated proteins were isolated by Acyl-RAC for Western blot analysis. (g and h) CHX chase assay of WT and C1167Y mutant NPC1. HEK-293T cells were set up and transfected with the indicated plasmids as in (a). On day 3, cells were cultured in complete medium, and treated with CHX (50 μg/mL) for indicated time before harvesting for Western blot analysis. Band intensities were quantified with ImageJ using Calnexin as an internal control (h; *n *= 3). (i and j) Fluorescence intensity profiles of NPC1-Flag and LAMP1-HA colocalization. On day 0, HeLa cells were set up. On day 1, cells were transfected with the indicated plasmids. On day 3, cells were harvested for immunostaining. Fluorescence intensity profiles of NPC1-Flag and LAMP1-HA signals along the indicated lines were generated using ZEN software (i). The percentage of NPC1-Flag-positive area overlapping with LAMP1-HA-positive lysosomal structures from 10 cells was quantified using ImageJ software (j). (k and l) Lysosomal fraction analysis of WT and NPC1-4CS mutant by immunoblotting. On day 0, HEK-293T cells were set up. On day 1, cells were transfected with the indicated plasmids. On day 2, cells were harvested for lysosomal fraction isolation using the lysosome-preserving subcellular fractionation protocol as described in the Methods. HA-LAMP1 was used as a lysosomal marker for lysosomal fraction enrichment. Lysosomal fractions and total cell lysates were analyzed by immunoblotting for NPC1 and LAMP1 (k). Cholesterol levels in the isolated lysosomal fractions were measured and normalized to the amount of lysosomal proteins (l; *n *= 3). (m and n) Filipin staining of WT and *NPC1*^−/−^ HeLa cells reconstituted with WT or NPC1-4CS. WT and *NPC1*^−/−^ HeLa cells were set up, transfected with the indicated plasmids. On day 2, cells were cultured in DMEM supplemented with 5% DFBS for 12 h, followed by the addition of 20 μg/mL LDL for an additional 12 h. On day 4, cells were harvested for immunostaining analysis with Filipin (m). Quantification was performed as in Fig. 1b (n; *n *= 50). (o) Cell viability assay of WT and *NPC1*^−/−^ HeLa cells reconstituted with WT or NPC1-4CS. WT and *NPC1*^−/−^ HeLa cells (*n *= 3 per condition) were set up, transfected with indicated plasmids, and treated as in Fig. 1c. Each value represents mean ± SEM (c, h, j, l, n, and o). *P*-values were calculated from two-tailed unpaired *t*-tests.

To determine whether DHHC13 directly catalyzes NPC1 palmitoylation, we performed an *in vitro* palmitoylation assay using purified DHHC13-HA and NPC1-Flag (Fig. 3d and e). DHHC13 specifically palmitoylated WT NPC1, but not the 4CS mutant or the C1160SC1167S mutant (Fig. 3e; Supplementary Fig. S2d). Together, these data identify DHHC13 as the palmitoyltransferase for NPC1 and establish Cys799, Cys800, Cys1160, and Cys1167 as four critical residues required for maintaining NPC1 palmitoylation.

We next examined the palmitoylation of disease-associated NPC1 mutant C1167Y. Compared with WT NPC1, the C1167Y mutant exhibited dramatically reduced palmitoylation (Fig. 3f). Similar to the 4CS mutant, the C1167Y mutant exhibited shorter half-life compared with WT NPC1 (Fig. 3g and h). These data indicate that the pathogenic C1167Y disrupted the palmitoylation and stability of NPC1.

We went on to examine the effect of palmitoylation on the subcellular localization of NPC1. As the 4CS and C1167Y mutants were not stable, we tried two strategies to enhance protein levels. First, we treated cells with bafilomycin A1, and found that the 4CS and C1167Y mutants were colocalized with LAMP1 (Supplementary Fig. S2f). However, as these two mutants were degraded in lysosomes, we could not draw a conclusion that palmitoylation of NPC1 did not affect lysosomal localization. We then increased the amount of the mutant plasmids in the transfection so that the levels of the 4CS and the C1167Y mutants were similar to those of WT NPC1 (Fig. 3g and h). As illustrated by both the colocalization and biochemical analysis, the lysosomal content of the 4CS and C1167Y mutants was dramatically decreased (Fig. 3i–k), indicating that palmitoylation is required for the lysosomal localization of NPC1. Importantly, when reintroduced into NPC1-deficient cells, neither the 4CS nor the C1167Y mutant was able to rescue lysosomal cholesterol accumulation (Fig. 3l–n), which was further confirmed by the AmB analysis (Fig. 3o).

### *Dhhc13* ^−/−^ mice exhibit aberrant cholesterol accumulation in multiple tissues

To further validate the results, we generated *Dhhc13*^−/−^ mice using the CRISPR-Cas9 system (Supplementary Fig. S3a). From week 4 to week 14, body weights of *Dhhc13*^−/−^ mice were consistently lower than those of WT mice, suggesting delayed development (Fig. 4a). Despite this, the relative tissue weights (as percentages of body weight) of the spleen, liver, brain, and kidney were significantly higher in the *Dhhc13*^−/−^ mice compared with those of WT controls (Fig. 4b). Cholesterol contents in the spleen, liver, brain, and kidney of *Dhhc13*^−/−^ mice were also significantly higher (approximately 1.5- to 2-fold) than those in WT mice (Fig. 4c–f). Filipin staining confirmed that cholesterol was accumulated in the lysosomes of primary hepatocytes in *Dhhc13*^−/−^ mice (Supplementary Fig. S3b and c).

**Figure 4:**
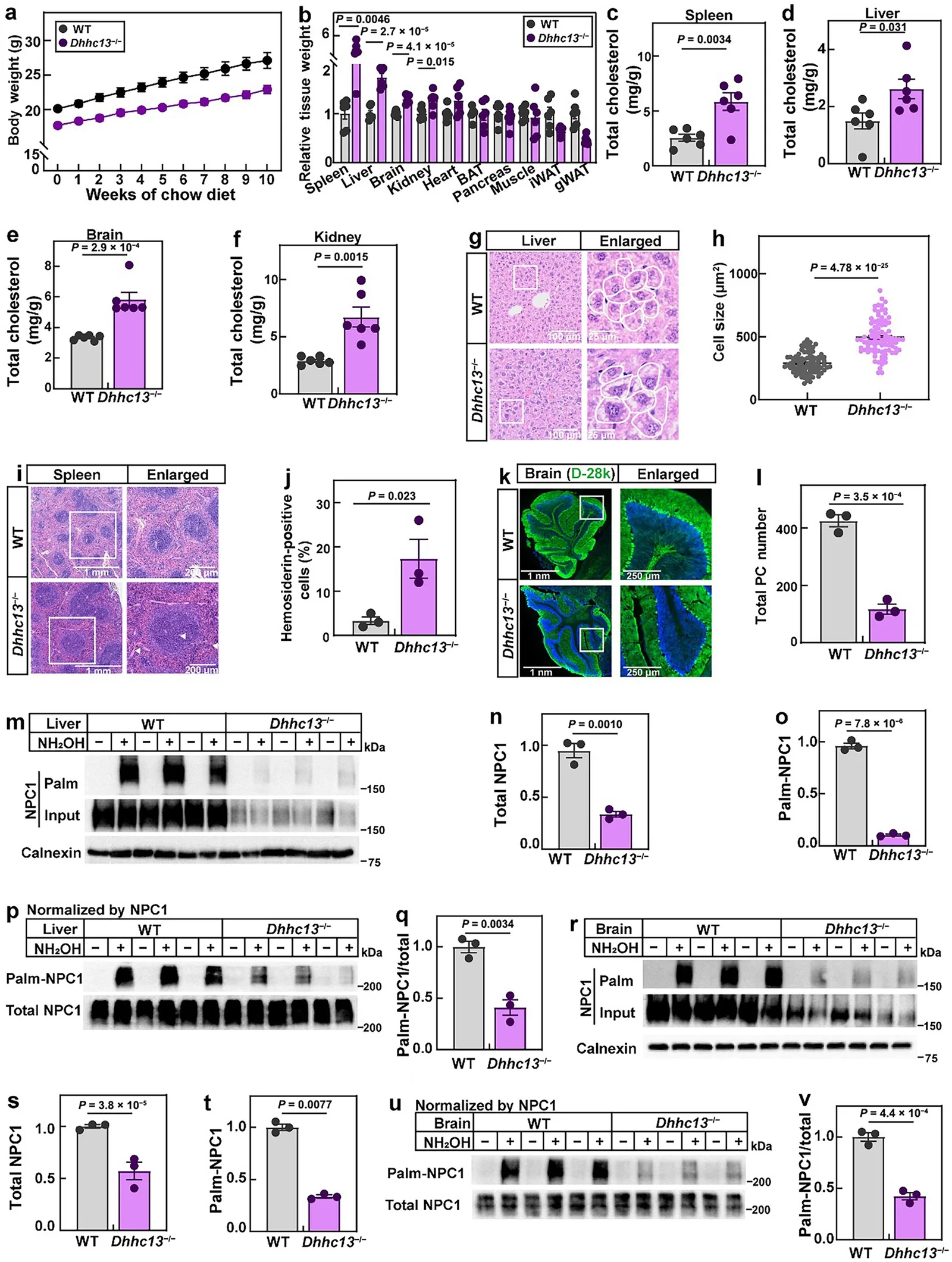
*Dhhc13* ^−/−^ mice exhibit aberrant cholesterol accumulation in multiple tissues. (a) Body weight monitoring of WT and *Dhhc13*^−/−^ mice on a chow diet. Body weights of WT and *Dhhc13*^−/−^ mice on a chow diet were monitored every week. Each value represents mean ± SEM of 6 mice. (b) Relative tissue weights of WT and *Dhhc13*^−/−^ mice. Tissues were dissected, blotted dry, and weighed immediately using an electronic analytical balance. (c–f) Total cholesterol content in the spleen, liver, brain, and kidney. Total cholesterol content in the spleen (c), liver (d), brain (e), and kidney (f) of 30-week-old WT and *Dhhc13*^−/−^ mice (maintained on a chow diet) was measured. (g−j) Hematoxylin and eosin (H&E) staining of mouse liver spleen tissues. H&E staining was performed on mouse liver (g) and spleen (i) tissues to observe morphological changes. Hepatocytes alterations (h) and the percentage of hemosiderin-positive cells in the spleen (j) were quantified using ImageJ. (k and l) Immunostaining of Purkinje cells with Calbindin-D-28K in the cerebellum. Mouse cerebellar tissues were immunostained for Calbindin-D-28K, a specific marker for Purkinje cells (k), and the total number of Purkinje cells (PCs) in the cerebellum was quantified (l). PC = Purkinje cell. (m–q) Western blot analysis of total and palmitoylated NPC1 in mouse livers. Total and palmitoylated proteins were isolated from the livers of 30-week-old WT and *Dhhc13*^−/−^ mice, followed by Western blot analysis (m). Band intensities of total NPC1 (n) and palmitoylated NPC1 (o) were quantified using ImageJ and normalized to Calnexin. Samples were normalized to NPC1 levels (p). Band intensities of normalized palmitoylated NPC1 (q) were quantified using ImageJ. (r–v) Western blot analysis of total and palmitoylated NPC1 in mouse brains. Total and palmitoylated proteins were isolated from the brains of 30-week-old WT and *Dhhc13*^−/−^ mice, followed by Western blot analysis (r). Band intensities of total NPC1 (s) and palmitoylated NPC1 (t) were quantified using ImageJ and normalized to Calnexin. Samples were normalized to NPC1 levels (u). Band intensities of normalized palmitoylated NPC1 (v) were quantified using ImageJ. Each value represents mean ± SEM of 6 mice (a−f). Each value represents mean ± SEM of 3 samples (h, j, l, n, o, q, s, t, and v). *P*-values were calculated from two-tailed unpaired *t*-tests.

Histological examination revealed that hepatocytes in *Dhhc13*^−/−^ mice were approximately twice the size of those in WT mice (Fig. 4g and h). The number of hemosiderin-positive cells was significantly increased (about 4-fold) in the spleens of *Dhhc13*^−/−^ mice (Fig. 4i and j). Furthermore, the number of Purkinje cells was significantly reduced (from 425 to 117) in the brains of *Dhhc13*^−/−^ mice (Fig. 4k and l).

Consistently, the levels of total NPC1 and palmitoylated NPC1 were significantly decreased in the spleen, liver, brain, and kidney of *Dhhc13*^−/−^ mice (Fig. 4m–o and r–t; Supplementary Fig. S3d–f and S3i–k). Even after normalization of total NPC1 levels, the palmitoylation of NPC1 was still dramatically decreased in the knockout mice (Fig. 4p, q, u, and v; Supplementary Fig. S3g, h, l, and m). These results further confirmed that DHHC13 functions through NPC1 to regulate cholesterol metabolism. Collectively, *Dhhc13*^−/−^ mice phenocopied *Npc1*^−/−^ mice with respect to these pathological features [44].

### Two clinically relevant DHHC13 mutants show reduced activity in palmitoylating NPC1

We next investigated whether clinically relevant DHHC13 mutants are associated with neurodegenerative diseases. Genome-wide association studies (GWAS) revealed that two mutants, Q173H and R451Q, were positively associated with neurodegenerative diseases (Fig. 5a). Structural analysis revealed that Gln173 is localized in the ANK domain, which is the substrate binding domain [45, 46], and Arg451 is adjacent to the DQHC (aa 453–456) active site (Fig. 5b). Immunoprecipitation analysis revealed that compared with WT NPC1, the Q173H indeed showed decreased interaction with NPC1 (Fig. 5c). Although R451Q retained its interaction with NPC1 (Fig. 5c), it exhibited markedly reduced autopalmitoylation (Fig. 5d), confirming that R451Q is a catalytically compromised mutant. When reintroduced into DHHC13-deficient cells, both Q173H and R451Q mutants showed dramatically reduced activity toward palmitoylating NPC1 (Fig. 5e), indicating that both are loss-of-function mutants.

**Figure 5:**
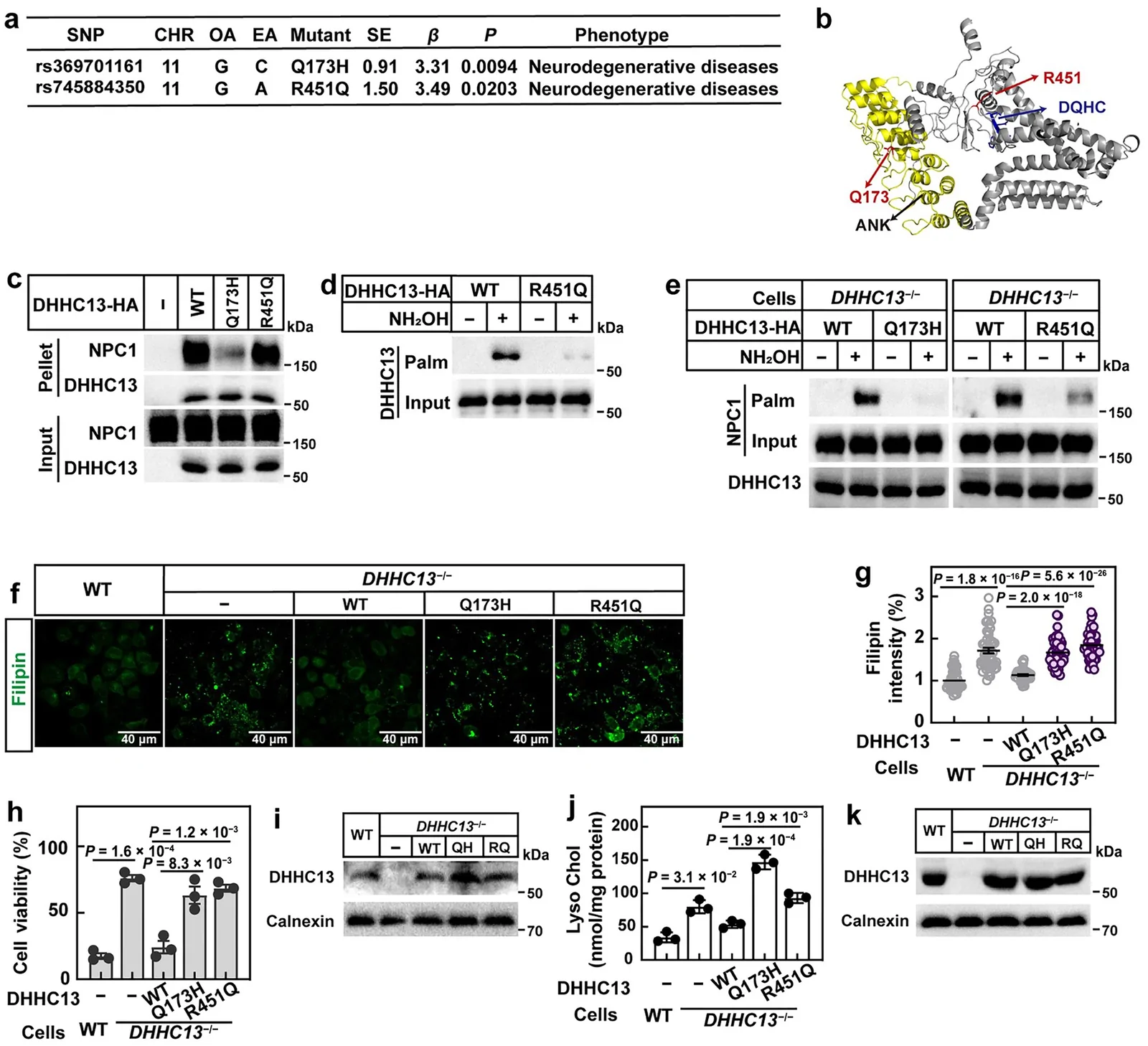
Two clinically relevant DHHC13 mutants show reduced activity in palmitoylating NPC1. (a) Meta-analysis of GWAS variants Q173H and R451Q in DHHC13 associated with neurodegenerative traits. Meta-analysis of GWAS summary statistics identified two DHHC13 (ZDHHC13) variants (Q173H and R451Q) associated with neurodegenerative disease-related traits. (b) Schematic representation of the DHHC13 structure. The locations of the Q173H and R451Q variants, as identified by text labels and arrows. (c) Co-immunoprecipitation assay of WT, Q173H, and R451Q DHHC13-HA with NPC1. On day 0, HEK-293T cells were set up and transfected with 2 μg of either WT, Q173H, and R451Q DHHC13-HA/pCDH-puro. On day 3, cells were harvested and subjected to Co-IP followed by Western blot analysis. (d) Acyl-RAC assay of WT and mutant DHHC13-HA in HEK-293T cells. HEK-293T cells were set up, and transfected with indicated plasmids. On day 3, cells were harvested for Acyl-RAC analysis. (e) Acyl-RAC assay of WT and mutant DHHC13 in WT and *DHHC13*^−/−^ HEK-293T cells. WT and *DHHC13*^−/−^ HEK-293T cells were set up, and transfected with the indicated plasmids. On day 3, cells were harvested for Acyl-RAC analysis. (f and g) Filipin staining of WT and *DHHC13*^−/−^ HeLa cells reconstituted with WT or mutant DHHC13. WT and *DHHC13*^−/−^ HeLa cells were set up, and transfected with the indicated plasmids. On day 3, cells were harvested for immunostaining analysis as in Fig. 1a (f). Quantification was performed as in Fig. 1b (g; *n *= 50). (h) Cell viability assay of WT and *DHHC13*^−/−^ HeLa cells. WT and *DHHC13*^−/−^ HeLa cells were set up, and transfected with the indicated plasmids. On day 3, cell viability were quantified following the protocol in Fig. 1c. (i) Western blot analysis of NPC1 protein levels in reconstituted HeLa cells. WT and *DHHC13*^−/−^ HeLa cells were set up, and transfected with the indicated plasmids. On day 3, cells were harvested for Western blot analysis. (j) Quantification of lysosomal cholesterol levels in reconstituted HeLa cells. WT and *DHHC13*^−/−^ HeLa cells were set up (*n *= 3), and transfected with the indicated plasmids. On day 3, cells were treated as in Fig. 3j to assess lysosomal cholesterol levels. (k) Western blot analysis of DHHC13 in reconstituted HEK-293T cells. WT and *DHHC13*^−/−^ HEK-293T cells were set up (*n *= 3), and transfected with the indicated plasmids. On day 3, cells were harvested for Western blot analysis. Each value represents mean ± SEM (g, h, and i). *P*-values were calculated from two-tailed unpaired *t*-tests.

Functionally, as illustrated by Filipin staining, direct measurement of lysosomal cholesterol content, and AmB analysis, when either mutant was re-introduced into *DHHC13*^−/−^ cells, they failed to reverse lysosomal cholesterol accumulation (Fig. 5f–k). These results further confirmed that the Q173H and R451Q mutants are pathogenic.

We then proposed a working model to summarize our findings (Fig. 6). In WT cells, DHHC13 palmitoylates NPC1 to promote the lysosomal targeting, stability, and cholesterol egress activity of NPC1. In cells lacking functional DHHC13, reduced NPC1 palmitoylation impairs its lysosomal targeting and promotes its degradation, leading to lysosomal cholesterol accumulation.

**Figure 6:**
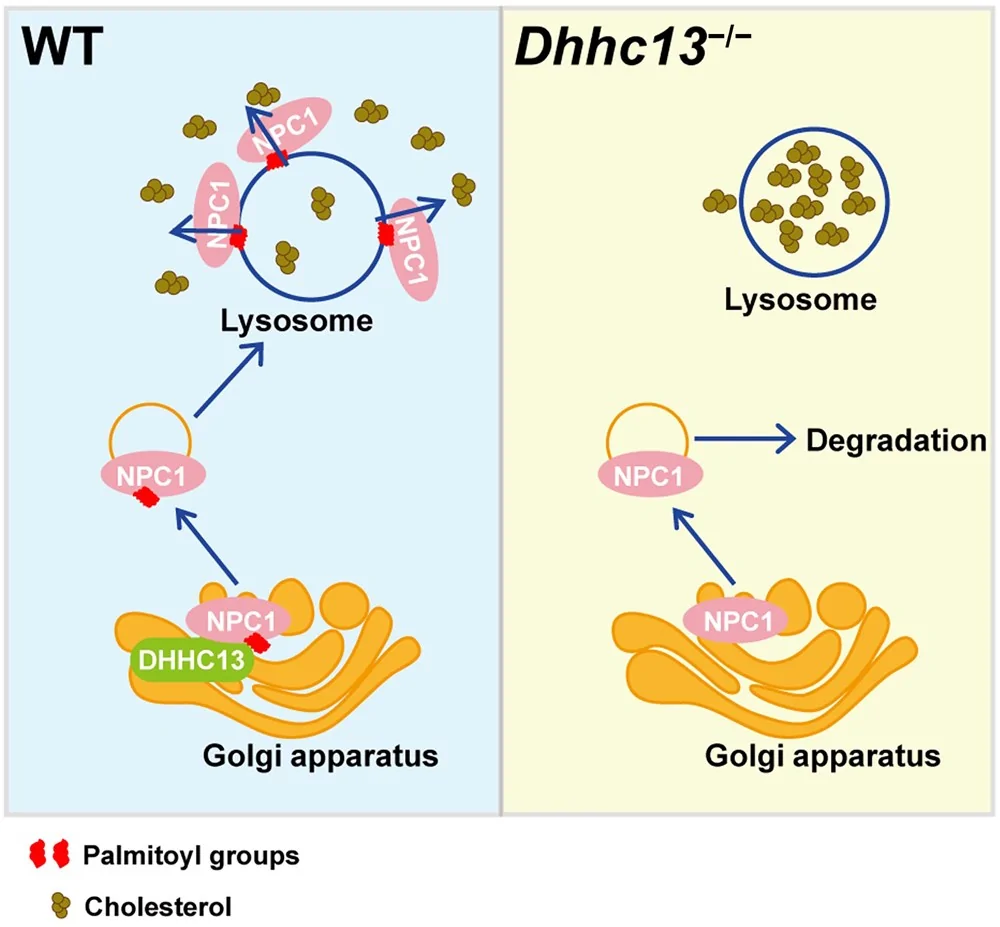
Working model. In WT cells, DHHC13 palmitoylates NPC1, with Cys799 and Cys800 serving as major palmitoylation sites and Cys1160 and Cys1167 acting as critical regulatory residues required for efficient palmitoylation. This modification promotes NPC1 lysosomal targeting, stability, and cholesterol egress. In cells lacking functional DHHC13, NPC1 palmitoylation is reduced, impairing its lysosomal targeting and promoting its degradation, thereby leading to lysosomal cholesterol accumulation.

## Discussion

Protein palmitoylation and cholesterol metabolism have long been known to intersect at the level of membrane microdomains, where palmitoylated proteins are enriched in a cholesterol-dependent manner [47]. However, whether protein palmitoylation reciprocally regulates cholesterol homeostasis has remained largely unexplored. Here, we establish a direct mechanistic link between these two fundamental biological processes by identifying DHHC13 as the palmitoyltransferase that modifies NPC1 and governs lysosomal cholesterol egress. DHHC13 palmitoylates NPC1 and controls its lysosomal localization, protein stability, and cholesterol transporting activity.

We demonstrate that *Dhhc13*^−/−^ resembled *Npc1*^−/−^ mice [44] in the NPC phenotypes, including cholesterol accumulation in the brain and liver, along with reduced Purkinje cell numbers. Notably, DHHC13 deficiency also caused systemic phenotypes including alopecia, osteoporosis, and amyloidosis [48], which are not all recapitulated in *Npc1*^−/−^ mice. This suggests that the role of DHHC13 extends beyond NPC1 palmitoylation, consistent with its involvement in palmitoylating diverse substrates such as peptidylarginine deiminases 3 (PADi3), transglutaminase 1 (TGM1), Unc-51-like autophagy-activating kinase 1 (ULK1), and membrane type 1-matrix metalloproteinase (MT1-MMP) [33–35, 49]. Nevertheless, the convergence of the two knockout models on lysosomal cholesterol accumulation positions DHHC13 as a major physiological regulator of this process.

Our findings identify DHHC13 as an NPC1 palmitoyltransferase and help clarify the controversy surrounding NPC1 palmitoylation. Previous studies proposed Cys97 [26] or Cys799 and Cys800 [27] as palmitoylation sites. In our assays, C97S had no detectable effect, whereas C799S/C800S markedly reduced NPC1 palmitoylation. On top of that, we found that Cys1160 and Cys1167 are also required for NPC1 palmitoylation. Notably, the NPC1 mutant C1167Y, identified in patients with NPC [42, 43], exhibited markedly reduced palmitoylation, shortened half-life, and diminished lysosomal localization, and failed to rescue cholesterol egress in NPC1-deficient cells. We therefore propose that its instability may result from the combined effects of impaired palmitoylation and mutation-induced structural alterations. To our knowledge, this is the first demonstration that a disease-associated NPC1 mutation exerts its pathogenic effect through impaired palmitoylation. This raises the intriguing possibility that palmitoylation-deficient NPC1 mutants may constitute a distinct subclass within the NPC1 mutational spectrum, with potential implications for genotype−phenotype correlations and therapeutic strategies.

However, whether Cys1160 and Cys1167 are directly modified remains to be determined. According to the reported cryo-EM structure of NPC1 [50], these two residues reside within a transmembrane helix, raising the possibility that their mutation may not eliminate direct palmitoylation but rather perturb local helix packing or conformational dynamics, thereby altering the accessibility of Cys799 and Cys800 to DHHC13. This interpretation is also constrained by a technical limitation of our approach: the Acyl-RAC assay enriches total S-palmitoylated proteins but does not distinguish which specific cysteine residues within a given protein are modified. Future studies employing site-specific palmitoylation detection methods, such as LC-MS/MS analysis of palmitoylated peptides, will be required to definitively determine whether Cys1160 and Cys1167 are directly palmitoylated or exert their effects through structural modulation.

Our findings support a role for palmitoylation in the intracellular sorting and steady-state lysosomal localization of NPC1. Loss of palmitoylation disrupts the localization of NPC1 in the lysosomes and causes its lysosomal degradation. Although we do not know the exact mechanism of how palmitoylation protects NPC1 from degradation, the plausible explanation is that palmitoylation may facilitate the partitioning of NPC1 into specific membrane microdomains during intracellular sorting or within lysosomal membranes. This possibility is supported by previous studies showing that S-palmitoylation can regulate membrane-cargo sorting and the partitioning of transmembrane proteins into ordered membrane domains [29, 51, 52]. Such membrane environments may help maintain an appropriate NPC1 conformation and promote productive interactions with lipids or partner proteins, thereby potentially reducing its susceptibility to quality-control-mediated degradation.

Furthermore, we identified two clinically relevant DHHC13 mutants, Q173H and R451Q, linked to neurodegenerative disorders through GWAS analysis. Both mutants failed to restore NPC1 palmitoylation or lysosomal cholesterol egress in DHHC13-deficient cells. These findings position DHHC13 as a potential susceptibility gene for cholesterol-related neurological disorders and suggest that compromised NPC1 palmitoylation may represent a shared pathogenic mechanism underlying a subset of these conditions.

Collectively, the DHHC13-NPC1 axis uncovered here provides a molecular framework for understanding how palmitoylation dynamically controls lysosomal cholesterol egress. Several important questions remain open for future investigation. First, it will be of interest to determine whether cholesterol accumulation itself feeds back to modulate DHHC13 expression or activity, which would establish a homeostatic regulatory loop. Second, given the broad substrate spectrum of DHHC13, it remains to be explored whether other cholesterol-related proteins, such as LDLR, Niemann-Pick C1-like 1 (NPC1L1), or acetyl-CoA acetyltransferase 1 (ACAT1), are also subject to DHHC13-mediated palmitoylation. Finally, the identification of palmitoylation-deficient clinical mutants in both NPC1 and DHHC13 raises the possibility that pharmacological modulation of this pathway, either by enhancing DHHC13 activity or by inhibiting NPC1 de-palmitoylation, could represent a therapeutic avenue for NPC disease and related cholesterol storage disorders. Our study thus lays the foundation for future efforts aimed at targeting this newly defined regulatory axis for therapeutic benefit.

## Limitations of the study

Several limitations should be acknowledged. First, given the broad substrate spectrum of DHHC13, we cannot exclude the possibility that some phenotypes observed in *Dhhc13*^−/−^ mice, particularly those unrelated to cholesterol metabolism, such as alopecia and osteoporosis, arise from hypopalmitoylation of substrates other than NPC1. Generation of NPC1 palmitoylation-deficient knockin mice (e.g., 4CS or C1167Y) will be required to definitively attribute the lysosomal cholesterol storage phenotype to impaired NPC1 modification. Second, as noted above, the Acyl-RAC assay did not distinguish which specific cysteine residues were directly palmitoylated; site-specific palmitoyl-proteomic approaches such as LC-MS/MS analysis of palmitoylated peptides will be necessary to resolve whether Cys1160 and Cys1167 are directly modified or exert their effects through structural modulation. Third, while we identified two clinically relevant DHHC13 mutants (Q173H and R451Q) with reduced catalytic activity in cell-based assays, whether these variants confer loss of function *in vivo* and directly contribute to neurodegeneration in patients awaits validation in animal models or patient-derived cells.

## Materials and methods

### Mice

WT C57BL6 mice were purchased at GemPharmatech. *Dhhc13*^−/−^ mice were generated by the CRISPR-Cas9 system. Two single-guide RNAs (sgRNAs) flanking exon 4–8 were designed. The genotyping primers were listed in Supplementary Table S1. *Dhhc13^+/^*^−^ mice were crossed with WT mice for two generations, and mated to generate WT and *Dhhc13*^−/−^ mice. All mice were housed in colony cages at 22°C with 12-h light/12-h dark cycles. The dark cycle began at 7 p.m. All animal studies were performed with the approval of the Institutional Animal Care and Research Advisory Committee at Fudan University. All mice used in this study were male on a C57BL/6 background. The chow diet (Xietong Organism, Nanjing, China) contains 12% calories from fat, 67.4% from carbohydrates, and 20.6% from protein.

### Plasmids

Full length cDNAs of mouse *Npc1* were cloned from a cDNA library prepared from the liver of a C57BL/6 mouse. Full length cDNAs of human *DHHC13* and *LAMP1* were cloned from a cDNA library prepared from HEK-293T cells. These genes were cloned into pCDH-EF1-MCS-IRES-Puro (pCDH-puro, System Biosciences) with indicated tags. The sgRNA and shRNAs were cloned into pX459 (Addgene, 62988) and pLKO.1 (Addgene, 10878), respectively. Site-directed mutagenesis was performed using commercial kits from New England Biolabs. The primers were listed in Supplementary Table S1.

### Cell culture

HEK-293T and HeLa cells were cultured in Medium A, which consisted of Dulbecco’s modified Eagle’s medium (4.5 g/L glucose) supplemented with 10% (v/v) FBS, 100 U/mL penicillin, and 100 mg/mL streptomycin, at 37°C in an atmosphere of 5% CO_2_.

### Generation of *DHHC13* ^−/−^ and *NPC1* ^−/−^ HEK-293T and HeLa cells

*DHHC13* ^−/−^ and *NPC1^−/−^* HEK-293T and HeLa cells were generated using the pX459 system [53, 54]. A sgRNA targeting the coding region of *DHHC13 or NPC1* was cloned into the pX459 CRISPR/Cas9 vector. The resulting plasmid was transfected into HEK-293T or HeLa cells, followed by puromycin selection (1 μg/mL) for 3 days. Surviving cells were subjected to single-cell cloning by limiting dilution in 96-well plates. Individual clones were expanded, and *DHHC13* disruption was verified by genomic analysis before downstream experiments. The sgRNA sequences used in this study were listed in Supplementary Table S1.

### Lentivirus production

Lentiviral particles were generated using a three-plasmid packaging system as previously described with modifications [55]. HEK-293T cells were co-transfected with transfer vectors containing the indicated genes (pCDH-puro) or shRNA sequences (pLKO.1), together with the packaging plasmids psPAX2 and pMD2.G. Viral supernatants were collected at 24 h and 48 h after transfection, pooled, concentrated, and stored at −80°C until use for transduction of target cells.

### Filipin staining for detection of intracellular free cholesterol

Intracellular free cholesterol was visualized by Filipin staining as previously described with minor modifications [39, 56]. Briefly, cells were cultured on glass coverslips and subjected to indicated treatments. For LDL loading experiments, cells were cultured in medium containing 5% delipidated FBS (DFBS) for 12 h, followed by incubation with LDL (20 μg/mL) for the indicated time points. Cells were then washed three times with phosphate‑buffered saline (PBS) and fixed with 4% paraformaldehyde for 30 min at room temperature.

Cells were then incubated with Filipin III solution (0.05 mg/mL; Sigma-Aldrich, SAE0088) for 1 h at room temperature in the dark. After extensive washing with PBS, cells were mounted with antifade mounting medium and imaged using fluorescence microscopy.

For colocalization analysis, cells were co-stained with antibodies against lysosomal markers as indicated. Images were acquired under identical exposure conditions between experimental groups. Filipin fluorescence intensity was quantified using Image J software. For each condition, at least 50 randomly selected cells from multiple independent fields were analyzed. The mean Filipin fluorescence intensity per cell was calculated and used as an indicator of intracellular free cholesterol accumulation.

### AmB-based lysosomal cholesterol egress assay

The cholesterol egress assay was performed based on a previously described method with some modifications [39]. Briefly, control or HeLa cells with knockdown of indicated DHHCs were cultured for 16 h in medium supplemented with 5% DFBS, 1 μmol/L lovastatin, and 10 μmol/L mevalonate. Subsequently, cells were incubated with medium containing LDL (30 μg/mL), lovastatin (1 μmol/L), and U18666A (1 μmol/L) for 4 h to promote LDL uptake and accumulation of LDL-derived cholesterol in late endosomes/lysosomes. Cells were treated with 1.5% methyl-β-cyclodextrin for 10 min, followed by washing twice with PBS. Cells were then incubated in DFBS-containing medium supplemented with lovastatin (1 μmol/L), mevalonate (10 μmol/L), and AmB (350 μg/mL) for 2–3 h. Cells were fixed with 4% paraformaldehyde and stained with 0.1% crystal violet for 8 min. After washing with tap water and air drying, crystal violet was extracted with 10% acetic acid under agitation. Absorbance was measured at 600 nm, and relative cell survival was calculated by normalizing to untreated control cells.

### Lysosomal fraction purification and cholesterol quantification

This assay was performed as previously described with some modifications [57]. First, control or HEK-293T cells with indicated treatment were lysed in KPBS buffer supplemented with protease and phosphatase inhibitor cocktail (MCE), and homogenized using a glass Dounce homogenizer. Subsequently, the homogenate was centrifuged at 3000 *g* for 5 min at 4°C. The supernatant was collected and incubated with HA beads (Smart-Lifesciences, SA068025) for 4 h at 4°C. The lysosomal fractions were eluted and dissolved in KPBS buffer containing 50 mmol/L DTT and 1% SDS, and incubated at 37°C for 1 h. An aliquot of 10 μL was taken for protein concentration determination using the BCA assay (Thermo Fisher Scientific, A55865). The remaining sample was mixed with methanol–chloroform solution (2:1, v/v) and incubated with gentle shaking at room temperature for 3 h. After centrifugation at 5000 *g* for 5 min at room temperature, the organic phase was collected and dried under a gentle stream of nitrogen. The dried samples were reconstituted in 1% Triton X-100 (dissolved in ethanol), and total cholesterol content was measured using a commercial total cholesterol assay kit according to the manufacturer’s instructions (FUJIFILM Wako, 293–93601).

### Isolation of palmitoylated proteins

S-palmitoylated proteins were enriched from HEK-293T WT and *DHHC13* knockout (*DHHC13*^−/−^) cells using an Acyl-RAC assay [8, 9]. Briefly, membrane fractions were prepared from WT and *DHHC13*^−/−^ HEK-293T cells, and membrane proteins were solubilized in Buffer A (100 mmol/L HEPES, pH 7.5, 1 mmol/L EDTA, and 2.5% SDS). Free thiol groups were blocked by incubation with 0.1% S-methyl methanethiosulfonate (MMTS) at 42°C for 60 min. After precipitation with pre-chilled acetone at −20°C for 2 h and washing with 70% cold acetone, protein pellets were subsequently dissolved in Buffer B (100 mmol/L HEPES, pH 7.5, 1 mmol/L EDTA, and 1% SDS), and S-palmitoylated proteins were captured with Acyl beads in the presence of either hydroxylamine (NH_2_OH, 0.21 mol/L) or NaCl (0.21 mol/L, negative control) at room temperature for 4 h with continuous rotation. Beads were extensively washed with Buffer B containing 8 mol/L urea (five washes, 5 min each) at 37°C, and eluted with Buffer B containing 50 mmol/L DTT at 37°C for 60 min. Acyl-RAC–enriched proteins were briefly separated by SDS-PAGE until they migrated approximately 1 cm into the resolving gel. The entire protein-containing region was excised without staining and directly subjected to in-gel digestion and mass spectrometry analysis using an EASY-nLC 1200 system (Thermo Fisher Scientific) interfaced with an Orbitrap Fusion Lumos mass spectrometer (Thermo Fisher Scientific) [58].

### Histological analysis

Mouse tissues were collected and fixed in 4% paraformaldehyde overnight at 4°C. Fixed tissues were processed, embedded in paraffin, and sectioned into 5-μm-thick slices. Sections were stained with hematoxylin and eosin (H&E) according to standard histological procedures. H&E-stained sections were examined by light microscopy. Liver morphology was evaluated by measuring hepatocyte size from randomly selected fields using Image J software. Splenic pathological changes were assessed based on H&E staining, and hemosiderin-positive cells were quantified from randomly selected areas [24]. Cerebellar sections were subjected to immunofluorescence staining with anti-Calbindin-D-28K antibody to evaluate Purkinje neuron numbers [25].

### *In vitro* palmitoylation assay

The *in vitro* palmitoylation assay was performed as previously described [59], with minor modifications. DHHC13-HA and tagged-FLAG WT or mutant NPC1 proteins were purified from HEK-293T cells using anti-HA and anti-FLAG affinity beads. Purified HA-DHHC13 and the indicated FLAG-NPC1 proteins were incubated in reaction buffer (50 mmol/L HEPES, pH 7.0, 200 mmol/L NaCI, 0.05 μmol/L DTT, 1 mmol/L EDTA, and 0.3 mmol/L DDM), 100 μmol/L palmitoyl-CoA, and 5 μmol/L ATP at 37°C for 1 h. Reactions lacking HA-DHHC13 were included as negative controls. NPC1 palmitoylation was measured using Acyl-RAC. Palmitoylated NPC1 and total NPC1 were detected by immunoblotting with an anti-FLAG antibody.

### Co-immunoprecipitation (Co-IP)

Immunoprecipitation was performed as previously described [60]. Briefly, HEK-293T cells were harvested and lysed in Buffer C (50 mmol/L Tris-HCl, pH 7.4, 150 mmol/L NaCl, 1% Triton X-100, and 0.1% SDS), supplemented with protease and MCE. After clarification by centrifugation, the soluble cell extracts were incubated with anti-FLAG M2 magnetic beads (Smart-Lifesciences, SA042100) or anti-HA beads (Smart-Lifesciences, SA068025) to capture the corresponding tagged proteins. Following incubation, the beads were washed extensively with Buffer D (50 mmol/L Tris-HCl, pH 7.4, 150 mmol/L NaCl, and 0.1% Triton X-100) for five times to remove nonspecifically bound proteins. The immunoprecipitated complexes were eluted by incubation with SDS loading buffer at 37°C for 1 h and subsequently analyzed by SDS-PAGE and Western blotting.

### Quantitative real-time PCR (qRT-PCR)

Total RNA was isolated from the samples using Trizol reagent as previously described [61]. The isolated RNA was reverse transcribed into complementary DNA (cDNA) using a reverse transcription kit (Toyobo, FSQ-301). Quantitative PCR was subsequently performed using SYBR Green-based detection chemistry (Yeasen, 11203ES08) on a real-time PCR platform. The relative abundance of target transcripts was calculated using the comparative Ct method. Expression levels were normalized to endogenous reference genes, including 36B4 or cyclophilin, as indicated. Primer sequences used for qRT-PCR analysis are provided in Supplementary Table S1.

### Immunofluorescence

We performed immunofluorescence as previously described [60]. Briefly, cells or tissue sections were fixed with 4% paraformaldehyde for 10–15 min at room temperature and washed three times with PBS. For permeabilization, samples were incubated with 0.1% Triton X-100 in PBS for 10 min at room temperature. After blocking with 5% bovine serum albumin (BSA) in PBS for 1 h, samples were incubated with primary antibodies overnight at 4°C. After washing with PBS, samples were incubated with appropriate fluorophore-conjugated secondary antibodies for 1 h at room temperature in the dark. Nuclei were counterstained with 4'-6-diamidino-2-phenylindole (DAPI), and samples were mounted using antifade mounting medium. Images were acquired using a Zeiss LSM 880 confocal laser scanning microscope or an Olympus SIM-Ultimate structured illumination microscopy system. Three-dimensional reconstruction was performed using Imaris 9.5 based on images acquired by Olympus SIM-Ultimate structured illumination microscopy.

The following primary antibodies were used: Filipin (1:50, Sigma-Aldrich, SAE0088), LysoTracker (Yeasen, 40738ES50), anti-Flag (Proteintech, 20543-1-AP), anti-HA (1:200, Roche, 11867423001), and anti-Calbindin D-28k (Proteintech, 14479-1-AP). The secondary antibodies used were: Donkey anti-Mouse IgG Alexa Fluor 488 (Thermo Fisher Scientific, A21202), Goat anti-Rat IgG Alexa Fluor 594 (Thermo Fisher Scientific, A11007), and Goat anti-Rabbit IgG Alexa Fluor Plus 555 (Thermo Fisher Scientific, A21429).

For protein localization analysis, fluorescence images were acquired under identical imaging conditions, and the spatial overlap between two fluorescent signals was quantified using Image J/Fiji software [29]. The overlapping area between two fluorescence channels was measured, and the percentage of overlapping fluorescence area relative to the indicated fluorescence signal was calculated to evaluate protein localization. Fluorescence intensity profiles along selected lines were generated using ZEN software (Carl Zeiss) to visualize the spatial distribution and coincidence of fluorescent signals.

### GWAS analysis

Summary-level GWAS data for the target phenotype were obtained from the publicly available whole-genome sequencing dataset of 490,640 UK Biobank participants [62]. For downstream analyses, summary statistics including single nucleotide polymorphism (SNP) identifiers, chromosome positions, effect alleles, effect sizes, standard errors, and association *P*-values were obtained from the published GWAS results. Before downstream analysis, SNP annotations and effect alleles were harmonized to ensure consistency in genomic coordinates and effect direction. Genetic variants associated with the target phenotype were identified based on the reported effect estimates and statistical significance. All statistical analyses and downstream data processing were performed using R software (version 4.5.2; RStudio Desktop, Posit.). Genetic association analyses, statistical calculations, and visualization were performed using relevant R packages. Regional association plots were generated using LocusZoom.

### Western blot analysis

The primary antibodies used in this study were as follows: anti-Flag (Proteintech, 20543-1-AP), anti-HA (1:200, Roche, 11867423001), anti-NPC1 (Abcam, EPR5209), anti-DHHC13 (Abcam, AB28759), and anti-CALNEXIN (1:1000, Proteintech, 10427-2-AP). Western blots were developed using the Champ Gel 5000 imaging system (SINSAGE).

### Statistics and reproducibility

Statistical analysis was conducted using a two-tailed paired *t*-test in Excel 2010 or one-way ANOVA, as appropriate. No adjustment for multiple comparisons was applied. All values are presented as mean ± SEM. Complete statistical information, including the precise number of cells, can be found in the figure legends. No data points were excluded from the analysis. Each experiment was independently repeated at least twice with independent biological samples.

## Supplementary Material

loag028_Supplementary_Data

## Data Availability

All data generated or analyzed during this study are included in this published article and its supplementary information files.

## Notes

S.C. and T.J.Z. hold the position of Editorial Board Member for *Life Metabolism*, and were blinded from reviewing or making decisions for the manuscript. The other authors declare that no conflict of interest exists.

Ethics approval

All animal studies were performed with the approval of the Institutional Animal Care and Research Advisory Committee at Fudan University.
