# Supplementary material for: Palmitoyltransferase DHHC13 controls lysosomal cholesterol egress through palmitoylating Niemann-Pick C1
Source: Life Metab. 2026 Sep 10;5(6):loag028. doi: 10.1093/lifemeta/loag028 (PMC13671750; doi:10.1093/lifemeta/loag028)
Supplement: Supplementary file 1 [file loag028_supplementary_data.pdf]

Supplementary material for

DHHC13 controls lysosomal cholesterol egress through palmitoylating NPC1

Xiao-Ting Men[1,‡], Xiao-Yu Bai[1,‡], Yin-Yue Zhao[1], Yu-Qing Huang[1], Ding-Yuan Huo[1], Yu-Xiao Jiang[2], Ming-Feng Xia[2], Haobin Ye[1], Linzhang Huang[1], Xingrong Du[1], Hong-Jie Qian[3], Shuai Chen[4], Qing Shi[1,*], Tong-Jin Zhao[1,5,*]

[1]State Key Laboratory of Genetics and Development of Complex Phenotypes, Shanghai Key Laboratory of Metabolic Remodeling and Health, Institute of Metabolism and Integrative Biology, Zhongshan Hospital, Fudan University, Shanghai 200438, China

[2]Department of Endocrinology, Zhongshan Hospital, Fudan University, Shanghai 200032, China

[3]Drug Clinical Trial Center, Shanghai Xuhui Central Hospital/Xuhui Hospital, Fudan University; Shanghai Engineering Research Center of Phase I Clinical Research and Quality Consistency Evaluation for Drugs, Shanghai 200237, China

[4]State Key Laboratory of Pharmaceutical Biotechnology and MOE Key Laboratory of Model Animal for Disease Study, Model Animal Research Center, School of Medicine, Nanjing University, Nanjing, Jiangsu 210008, China

[5]State Key Laboratory of Metabolic Dysregulation & Prevention and Treatment of Esophageal Cancer, Innovation Center of Basic Research for Metabolic-Associated Fatty Liver Disease, Ministry of Education of China, Tianjian Laboratory of Advanced Biomedical Sciences, Academy of Medical sciences, Zhengzhou University, Zhengzhou, Henan 450001, China

‡These authors contributed equally to this work.

*Corresponding authors. Institute of Metabolism and Integrative Biology, Fudan University, Shanghai 200433, China. E-mail: shiqing_fdu@fudan.edu.cn (Q.S.); zhaotj@fudan.edu.cn (T.J.Z.)

Running head: DHHC13 palmitoylates NPC1 to control cholesterol egress

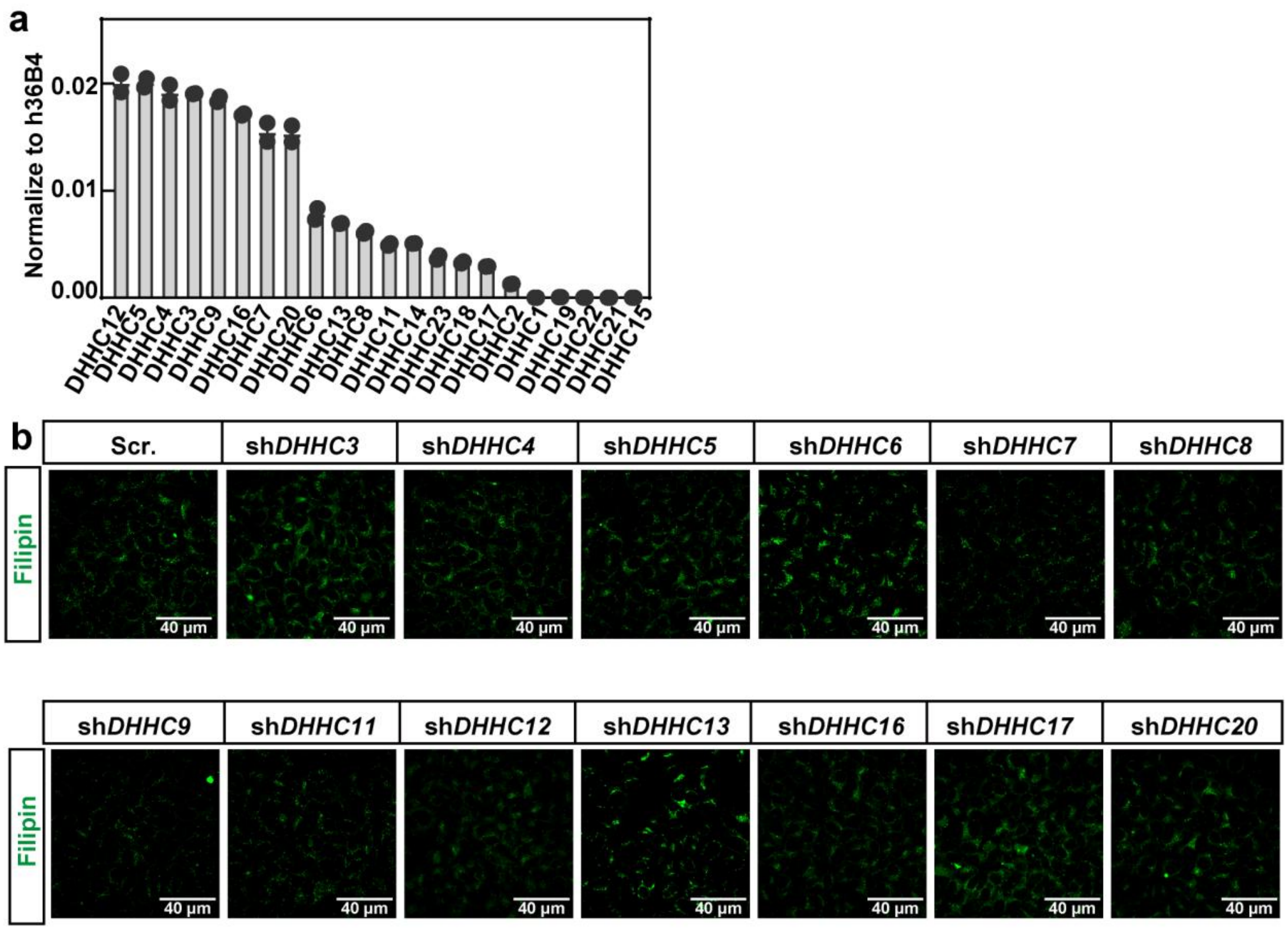

Supplementary Figure S1 DHHC13 is required for cholesterol egress from lysosomes. (a) Total RNA was extracted from harvested HeLa cells, and the expression levels of DHHC family proteins were determined using qRT-PCR (*n* = 3). (b) HeLa cells were seeded and treated as described in Fig. 1a. On day 1, cells were transduced with scrambled shRNA or specific shRNAs targeting DHHC family members (sh*DHHC3–9*, *11–13*, *16*, *17*, and *20*). On day 5, the cells were cultured in DMEM supplemented with 5% delipidated FBS (DFBS) for 12 h, followed by the addition of 20 μg/mL LDL for 12 h before harvesting for immunofluorescence analysis.

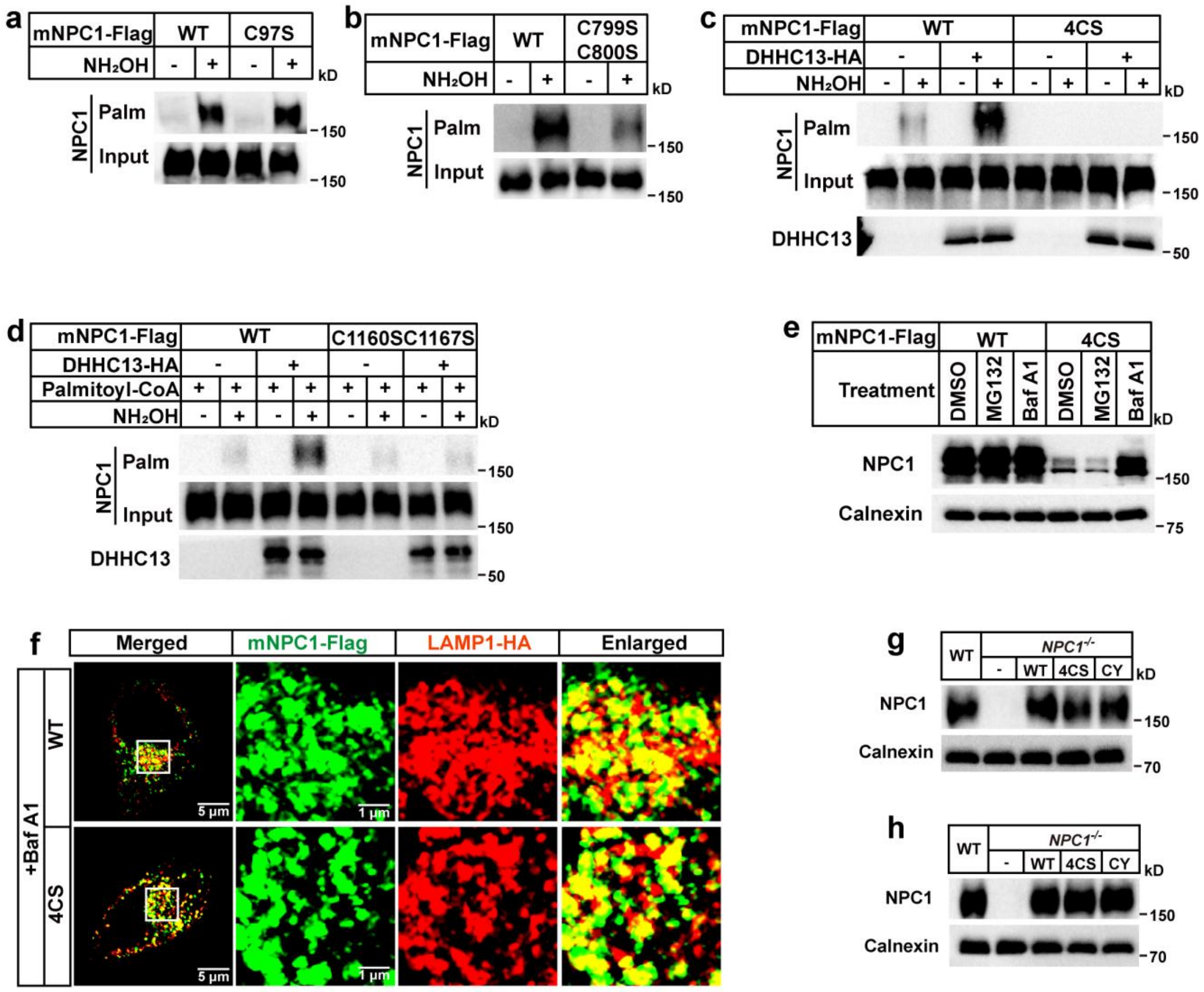

Supplementary Figure S2 Palmitoylation of NPC1 at Cys799, Cys800, Cys1160, and Cys1167 is required for its activity in lysosomal cholesterol egress. (a) On day 0, HEK-293T cells were set up at 2 × 10^5 cells per 6-cm dish. On day 2, cells were transfected with 2μg of either WT or C97S of mNPC1-FLAG/pCDH-puro. On day 3, total proteins were extracted for Acyl-RAC assay and subjected to Western blot. (b) On day 0, HEK-293T cells were set up at 2 × 10^5 cells per 6-cm dish. On day 2, cells were transfected with 2μg of either WT or C799, 800S of mNPC1-FLAG/pCDH-puro. On day 3, total proteins were extracted for Acyl-RAC assay and subjected to Western blot. (c) On day 0, HEK-293T cells were set up. On day 1, cells were transfected with indicated plasmids. On day 3, cells were harvested for Acyl-RAC assay and subjected to Western blot. (d) On day 0, HEK-293T cells were set up and transfected with

indicated plasmids. On day 3, cells were harvested and and the resulting DHHC13-HA and NPC1-WT/2CS-FLAG proteins were purified for the *in vitro* palmitoylation protocol. (e) On day 0, HEK-293T cells were set up at 2 × 10^5 cells per 6-cm dish. On day 2, cells were transfected with 2μg of either WT or 4CS of mNPC1-FLAG/pCDH-puro. On day 3, cells treated with MG132 (20 μg/mL) or Bafilomycin A1 (100 nmol/L) for 12 h. Proteins were extracted and subjected to Western blot. (f) On day 0, HeLa cells were set up as in Fig. 2k. On day 1, cells were transfected with indicated plasmids and treated with Bafilomycin A1 (100 nmol/L) for 12 h. On day 3, cells were harvested for immunostaining. (g and h) WT and *NPC1*$^{-/-}$ HeLa or 293T cells were set up, transfected with the indicated plasmids. On day 3, cells were harvested for Western blot.

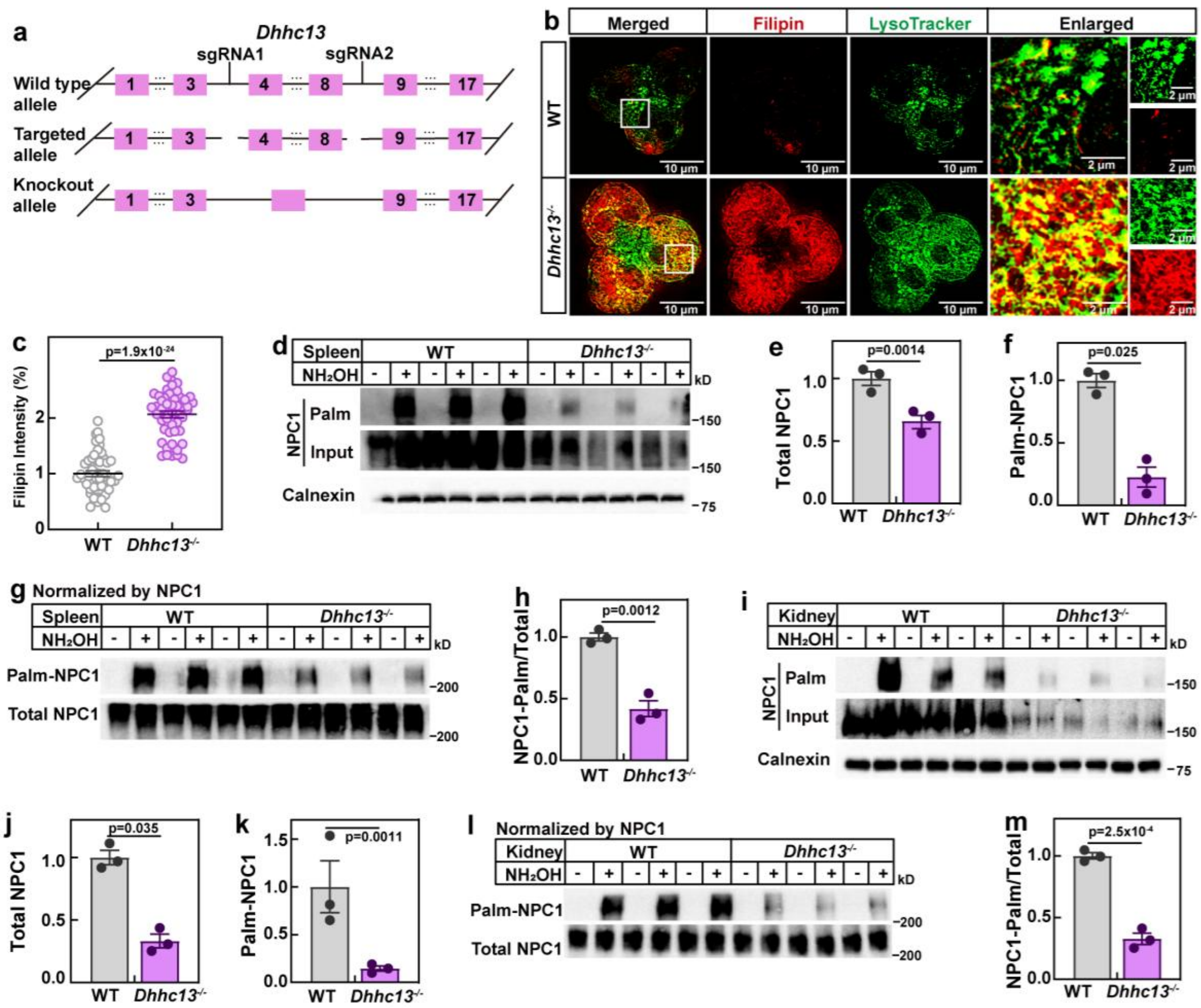

Supplementary Figure S3 *Dhhc13*$^{-/-}$ mice exhibit aberrant cholesterol accumulation in multiple tissues.

(a) Schematic illustration of the generation of *Dhhc13*$^{-/-}$ mice. (b and c) Primary hepatocytes isolated from WT and *Dhhc13*$^{-/-}$ mice were cultured *in vitro* for 6 h to promote cell attachment, stained with LysoTracker for 30 min, then harvested for immunostaining analysis with Filipin and Lysotracker. Quantification of Filipin is shown in (c), $n = 50$. (d–h) Total and palmitoylated proteins were isolated from the spleen of 30-week-old WT and *Dhhc13*$^{-/-}$ mice, followed by Western blot. Band intensities of total NPC1 (e) and palmitoylated NPC1 (f) were quantified using ImageJ and normalized to Calnexin. Samples were normalized to NPC1 expression levels (g). Band intensities of normalized palmitoylated NPC1 (h) were quantified using ImageJ. (i–m) Total and palmitoylated proteins were isolated from the kidney of 30-week-old WT and

Dhhc13$^{-/-}$ mice, followed by Western blot. Band intensities of total NPC1 (j) and palmitoylated NPC1 (k) were quantified using ImageJ and normalized to Calnexin. Samples were normalized to NPC1 expression levels (l). Band intensities of normalized palmitoylated NPC1 (m) were quantified using ImageJ. (e, f, h, j, k, and m) Each value represents mean ± SEM of 3 mice. *P*-values were calculated from two-tailed unpaired *t*-tests.

Supplementary Table S1. Primers used in the study.

Primers	Sequences
**(a) Genotyping sequences**	
Dhhc13-KO-1	ACCAGAGATTTGTCAGATGCTGTCA
Dhhc13-KO-2	TCCCTCATGCCTTAGAGGGATT
**(b) Targeted sequences of shRNAs and sgRNAs**	
h*DHHC13*-sgRNA-1	CACCGCTGGCCATACCTTAACATTC
h*DHHC13*-sgRNA-2	CACCGAAAATGTGTCGCTTCTTCAT
h*NPC1*-sgRNA-1	CACCGCTTCTCTGCCAACTATT
h*NPC1*-sgRNA-2	CACCGTTGACCTCGTGATCCC
sh*DHHC3*	CCGGGCTTTGAAGAAGATTGGACAACTCGAGTT GTCCAATCTTCTTCAAAGCTTTTTG
sh*DHHC4*	CCGGCATGGATTGCTTCATTACCTTCTCGAGAAG GTAATGAAGCAATCCATGTTTTTG
sh*DHHC5*	CCGGGGAAGCATTAGTGTTGACTGGCTCGAGCCA GTCAACACTAATGCTTCCTTTTTG
sh*DHHC6*	CCGGGGTTTACGATACTGGTTATATCTCGAGATAT AACCAGTATCGTAAACCTTTTTG
sh*DHHC7*	CCGGGGGTTATTACATGGGTTATTCCTCGAGGAAT AACCCATGTAATACCCTTTTTG
sh*DHHC8*	CCGGCAAAGCCTTAACCTTTGCTTTCTCGAGAAA GCAAAGGTTAAGGCTTTGTTTTTG
sh*DHHC9*	CCGGGTCTGTGATGGTGGTGAGAAACTCGAGTTT CTCACCACCATCACAGACTTTTTG
sh*DHHC11*	CCGGCCACCTTTGAGTATCTCATTACTCGAGTAAT GAGATACTCAAAGGTGGTTTTTG
sh*DHHC12*	CCGGTTCATCTCCTCACACCGCATCCTCGAGGAT GCGGTGTGAGGAGATGAATTTTTG
sh*DHHC13*	CCGGGTTTAATCCTTCTCTCAATGCTCGAGCATTG AGAGAAGGATTAAACTTTTTG
sh*DHHC16*	CCGGCCATCTGTAAGAAGTGCATTTCTCGAGAAA TGCACTTCTTACAGATGGTTTTTG
sh*DHHC17*	CCGGCCACAAGACAAGGCCATCTATCTCGAGATA GATGGCCTTGTCTTGTGGTTTTTG
sh*DHHC20*	CCGGGCAATGCGCTGGTAATTCTGGCTCGAGCCA GAATTACCAGCGCATTGCTTTTTG
**(c) Primers to generate different constructs**	
mNPC1-WT-F	ATGGGTGCGCACCACCCGGC
mNPC1-WT-R	AAAATTGAGGAGCCGTTCTC
mNPC1-C97S-F	CCTGTCCAGGTCTCCGTCATGTTTT
mNPC1-C97S-R	AAAAACATGACGGAGACCTGGACAG
mNPC1-C799S-F	GACATCCTGTCCTGTGTCAGAGGCG

mNPC1-C799S-R	CGCCTCTGACACAGGACAGGATGTC
mNPC1-C800S-F	GACATCCTGTgCTCTGTCAGAGGCG
mNPC1-C800S-R	CGCCTCTGACAGAGcACAGGATGTC
mNPC1-C799,800S-F	GGACATCCTGTCCTCTGTCAGAGGCGCTGACGAC GGACAA
mNPC1-C799,800S-R	TCTGACAGAGGACAGGATGTCCAGATGGTTTTTC TCTTGC
mNPC1-C1160S-F	TGGTGATGAGCTCCGGCATTTCTGT
mNPC1-C1160S-R	ACAGAAATGCCGGAGCTCATCACCA
mNPC1-C1167S-F	TGTGGAGTTCTCCAGCCATATAACG
mNPC1-C1167S-R	CGTTATATGGCTGGAGAACTCCACA
mLAMP1-WT-F	ATGGCGGCCCCCGGCAGCGC
mLAMP1-WT-R	TAGTCTGGTAGCCTGC
hDHHC13-WT-F	ATGGAGGGGCCGGGGCTG
hDHHC13-WT-R	TCATACTGAGCGAAGAACC
hDHHC13-Q173H-F	GGACACAGTGTGAATATGACAGATGTAAA
hDHHC13-Q173H-R	ACTGTGTCCCTTTGAGATGAGATATGCTAT
hDHHC13-R451Q-F	TGTGGCTCAATATGATCAACACTGCCTGTGGAC
hDHHC13-R451Q-R	ATATTGAGCCACACAGCAGTTGCATACAT
**(d) Quantitative real-time PCR primers**	
h*36B4*	TGCATCAGTACCCCATTCTATCA
	AAGGTGTAATCCGTCTCCACAGA
h*DHHC3*	AGCATCATCAACGGAATTGTGT
	CCGAATGCACCGCTTACAAAC
h*DHHC4*	TGATGGGTCTTGTTCTTATCTGC
	AGGTGTGGTTTCTCGTATGGAA
h*DHHC5*	CACCTGCCGCTTTTACCGT
	CGGCGACCAATACAGTTATTCAC
h*DHHC6*	AGTCTGCCAAGCATACAAGGC
	CCAGTGGTGCTAAAAGGAGAAAC
h*DHHC7*	CCCAAAGGAAACGCTACGAAA
	CGCGCTCGGGTTTAATACAG
h*DHHC8*	CTGTTCCCGTCTACAATGGCA
	GACCTGGATACCTCGCACAT
h*DHHC9*	CCTGGGTGGGGAATTGTGTT
	ACGACGGACCAGAGTGTAAAG
h*DHHC11*	GGTGCAGACCCTGATAGTCG
	GCACGTATGGATCTTTCCTCAC
h*DHHC12*	GTGCTGACCTGGGGAATCAC
	CTGCACATTCACGTAGCCA
h*DHHC13*	GGCCTGACCTCCCATGAGA
	CTGGCTGGGTGAAAGACCAT
h*DHHC16*	TGATGCTGCCTTTGAGCCTGTC

	GCACTGAGTAGGTTCGGAGGAT
h*DHHC17*	GATGTACGGCAACCGGACAAA
	TGATCCACAATAGCACCTTTCG
h*DHHC20*	TGTTGTTGACCCACATGGTTG
	GAGGCCCTCACTAAGGCAA
